# What do expectant mothers need to know about oral health? A cohort study from a London maternity unit

**DOI:** 10.1038/bdjopen.2017.4

**Published:** 2017-03-24

**Authors:** Patricia Nunes Correia, Aishah Alkhatrash, Catherine Ethel Williams, Annette Briley, Jenny Carter, Lucilla Poston, Marie Therese Hosey

**Affiliations:** 1 Paediatric Dentistry, Population and Patient Health Division, King’s College London Dental Institute, London, UK; 2 The Dental Centre, St Thoma’s Hospital, London, UK; 3 Division of Women’s Health, Women’s Health Academic Centre, King’s College London, London, UK

## Abstract

**Objective:**

To determine the oral health knowledge of pregnant women and to report their future plans to provide dental care for their expected child.

**Design and setting:**

Prospective cohort study; Ultrasound maternity services at St Thomas’ Hospital, London, 2014. Pregnant women attending for a routine ultrasound scan completed a questionnaire.

**Results:**

Women did not know that milk, dried fruit or fruit juices can cause caries. Most women knew about the benefit of fluoridated toothpaste, dental floss and sugar-free chewing gum, but only a minority knew about fluoride varnish. Most pregnant women planned to read or seek advice before purchasing their child’s first toothpaste. There was no difference regarding knowledge of prevention tools (diet and fluoride supplements) for dental caries (*P*>0.05) between first-time mothers and those who had children already. Though the latter knew more about toothpaste dose and timing of starting toothbrushing (*P*<0.05).

**Discussion:**

Oral health knowledge among pregnant women was deficient with respect to the cariogenicity of prolonged night-time milk feeding, dried fruits and fruit juice consumption. There was also limited knowledge of the benefit of fluoride varnish and timing of starting toothbrushing.

**Conclusions:**

Oral health knowledge amongst pregnant women is still deficient in many aspects. In this study population the need to improve maternal knowledge was shown.

## Introduction

Early childhood caries (ECC) is the most prevalent disease of childhood in the world. It constitutes, therefore a major challenge to public health. Early childhood caries is defined by the European Association of Paediatric Dentistry as ‘the occurrence of any sign of dental caries on any tooth surface during the first 3 years of life’^1^ and as ‘the presence of one or more decayed, missing (due to caries) or filled tooth surfaces in any primary tooth in a child 71 months of age or younger’.^2^ There is an association between ECC and the mother’s education attainment and socioeconomic status.^3,4^ Understandably, ‘infants’ oral health is highly dependent on their mothers’ motivation and ability to undertake the tasks required for oral care’.^5^ Equally determinant is the mother’s dietary habits and food choices when catering for their children.^6–8^ A strong campaign towards healthy eating has been promoted by the National Health Service (NHS) in the UK; however new food labels fail to inform consumers about the impact of food on teeth.

Early childhood caries is a highly complex disease, involving complex host–diet–microbe interactions.^7^ Day-time bottle feeding on demand, prolonged nocturnal bottle-feeding or breastfeeding, from 12 months of age and consumption of dried fruit and fruit juices between meals have all been linked to ECC.^9–13^ According to the WHO ‘breastfeeding is recommended up to 6 months of age and up to 2 years of age with complementary foods’.^14^ It is likely that prolonged and unrestricted milk feeding has an influence in caries development rather than being its sole cause.^15,16^

The accurate assessment of the knowledge and beliefs of parents regarding their children’s oral health can help create a tailored preventive program.^17,18^ Although there are many studies addressing the oral health-related knowledge of the mother/parents of preschool children,^17,19,20^ very few have examined the knowledge of pregnant women. The aims of this study were to determine the oral health knowledge and routine oral care habits of pregnant women and to report on their plans for the oral health of their baby.

## Materials and methods

A prospective cohort design was utilised and a pragmatic sample obtained. The participants were pregnant women attending for the routine 18–21 week ultrasound scan (anomaly scan) at St Thomas’s Hospital, in London.

The eligibility criteria were English speaking pregnant women, aged 18 years or older. A participant information sheet and verbal explanation was given to prospective participants and consent was obtained prior taking part in the study. The exclusion criteria were unwillingness to participate, not speaking/reading English, younger than 18 years of age or attending the ultrasound department for other scans. A self-report questionnaire was administered to participants. This contained questions from the UK Adult Dental Health Survey (ADHS) 2009, as well as specific questions relating to pregnancy, such as oral health habits during pregnancy, general oral health knowledge and oral health plans for their unborn baby. The questionnaire contained different topics entitled: ‘About me’, ‘Tooth decay and gum disease prevention’, ‘Delivery of oral health advice during this pregnancy’, ‘About my children’s teeth’, as well as optional questions on ‘Age’ and ‘Ethnic background’. The questionnaire had been discussed and improved by specialists within Dental Public Health, Midwifery (Research and Antenatal Education expertise) and Paediatric Dentistry. The pregnant women were asked to complete the questionnaire, in the waiting area, before their scan. As a token of appreciation, the participants were given a toothpaste sample. The data were entered into an SPSS database. Descriptive statistics such as mean and standard deviation (s.d.), frequency and percentage were calculated for continuous and categorical variables. Chi-square test was used to compare the knowledge of first-time mothers against those who had children already. The level of significance used was 0.05.

Ethical approval was obtained from a National Research Ethics Committee South West—Exeter (13/SW/0026) and Research and Development Department at Guy’s Hospital, prior to commencing the study (RJ113/N063).

## Results

**Figure 1:**
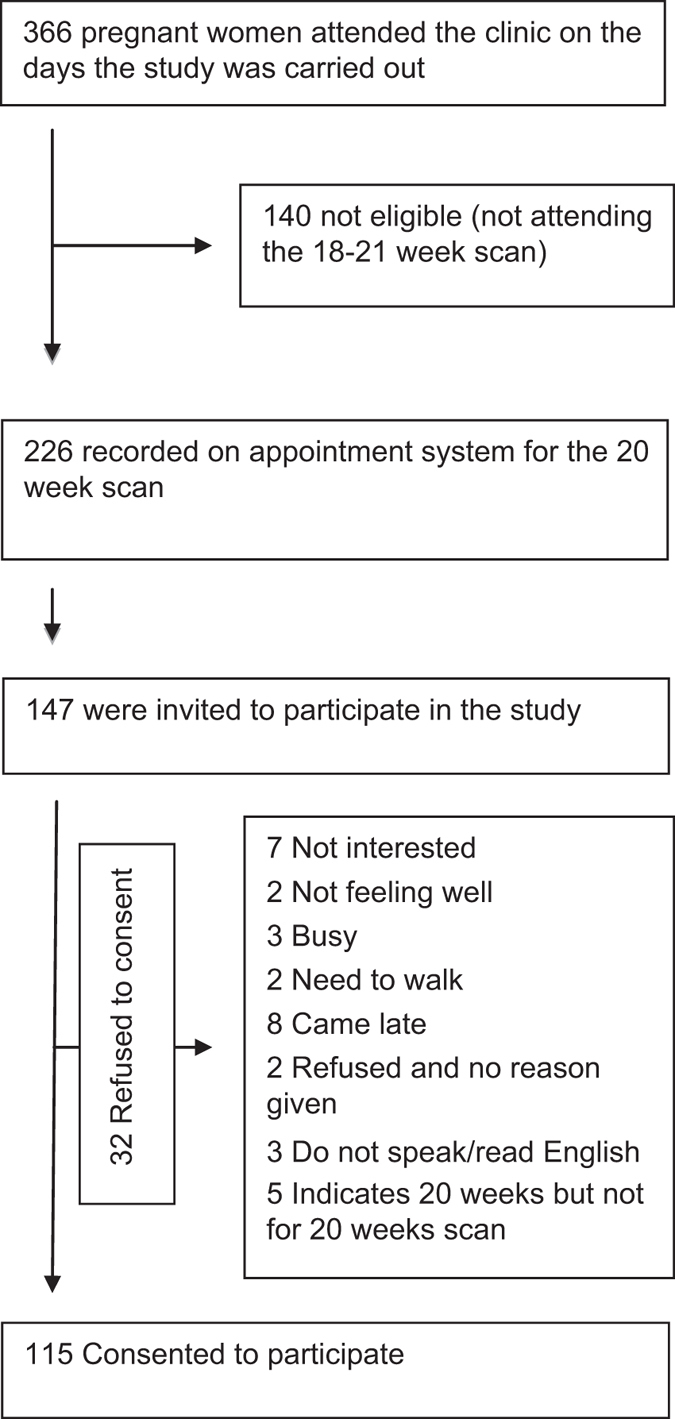
Recruitment flow chart.

From the 147 pregnant women approached, 115 women agreed to participate and met the inclusion criteria. The flow chart is shown in Figure 1.

The pregnant women’s demographics are shown in Table 1. In respect to self-reported ethnicity: 44% of the participants were White, 20% were Black, 4% were Mixed race, 7% were Asian, 5% belonged to other Ethnic groups and 20% did not respond.

Twelve per cent of participants rated their own oral health as very good, 61% reported that their oral health had not changed during pregnancy and 24% said it had deteriorated since their pregnancy.

More than a third (35%) of expectant mothers had not received oral health advice during their pregnancy. The others reported that they had received oral health advice from their dentist (30%), from midwives (21%) and from the general medical practitioner (7%). A number of them used external sources such as magazines (8%) and the internet (7%). The majority welcomed the idea of receiving oral health advice (57%), whereas the remaining were either ‘not interested’ (25%) or ‘not sure’ (11%).

Regarding oral health practices and behaviour, the weekly frequency of cariogenic food and drinks is recorded in Table 2.

**Figure 2:**
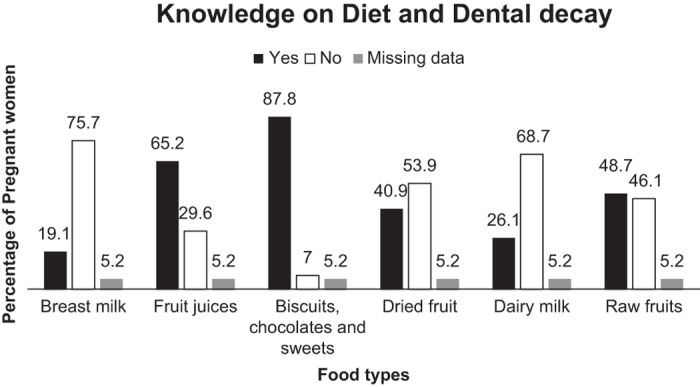
Knowledge of diet and dental decay.

The majority of participants knew that biscuits, chocolate and sweets (87.8%), and fizzy drinks (85.2%) caused caries. Nineteen per cent of them knew that breast milk could cause caries and 26% linked dairy milk to caries. Over half of them knew that fruit juices can cause caries, but only 41% knew that dried fruit is cariogenic. This is further detailed in Figure 2.

Their responses regarding oral health practices are presented in Table 3.

**Figure 3:**
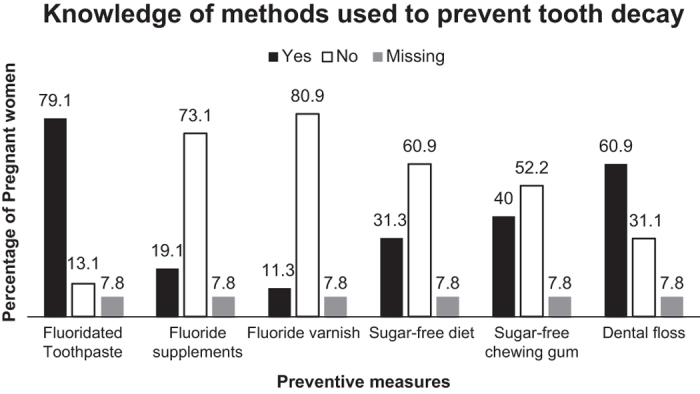
Knowledge of methods of prevention of dental caries.

The knowledge of caries prevention measures varied among the participants and this can be seen in Figure 3.

Regarding the plans that the expectant mothers had to care for their children’s teeth, 30 participants (26.1%) reported that they had not thought about the timing of cleaning their children’s teeth and 9 (7.8%) were uncertain. Fifty-seven of them (49.6%) planned to start brushing their baby’s teeth as soon as the first tooth comes through, 23 (20%) planned to wait for advice from a Health Professional, 16 (13.9%) were not sure, and 13 (11.3%) planned to do this when the baby started eating solids. Fifty women (43.5%) reported that they planned to ask or read before buying toothpaste for their child. Thirty-two (27.8%) were planning to use any children’s toothpaste and 26 (22.6%) said that they were ‘not sure’. A much lower number (8) of them planned to use toothpaste with 1,000 p.p.m. fluoride (7%) and two (1.7%) were planning to use an adult’s toothpaste. Two (1.7%) were not planning to buy toothpaste.

**Figure 4:**
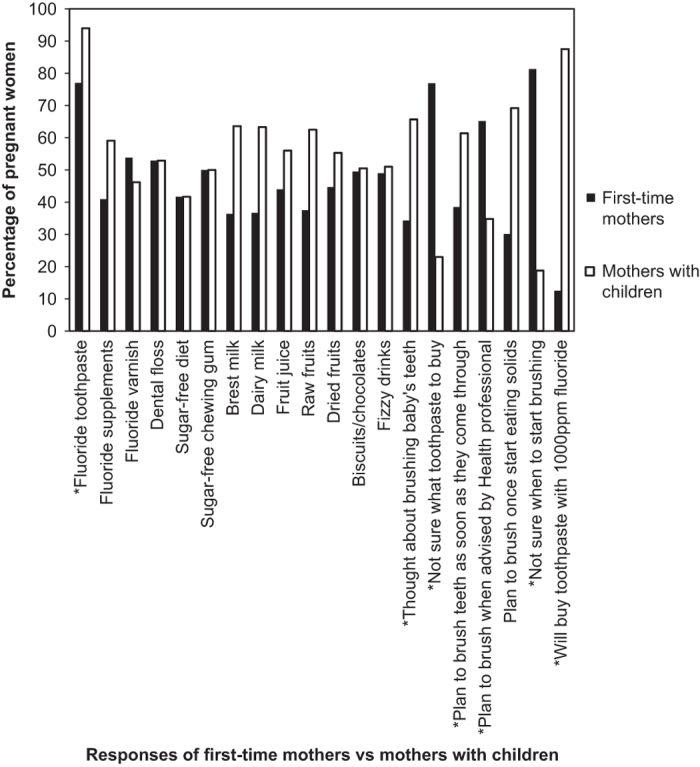
Percentage of responses on prevention between first-time mothers and mothers that already have children. *P<0.05.

When comparing knowledge among first-time mothers (*n*=58) and those with children already (*n*=56), no difference was noted regarding diet knowledge (Figure 4). However, those with children already were more likely to know that fluoride toothpaste prevented caries (*P*<0.02). Over seventy five per cent of the first-time mothers were less sure of which toothpaste to buy (*P*<0.001). Similarly, when asked whether they had plans to brush their children’s teeth, positive responses of first-time mothers were significantly lower than those from women with children (*P*<0.001) as were their answers regarding when to start brushing their children’s teeth (*P*<0.03), Figure 4.

Dental attendance was related to the knowledge of the preventive role of dental floss and sugar-free chewing gum: regular attenders were more knowledgeable than irregular attenders (*P*<0.012). Those who reported having received oral health advice in the past knew that dried fruits are cariogenic (*P*=0.016). Mothers who reported toothbrushing twice a day or more knew that milk and fruit juices can be cariogenic (*P*<0.05).

## Discussion

To our knowledge, this is the first study conducted in the UK assessing pregnant women’s oral health knowledge in relation to their children’s oral health needs. This study demonstrated a deficient knowledge in oral health in our sample of pregnant women. There is evidence to show that prevention of ECC is best initiated during pregnancy.^21–23^ The provision of social networks to support new mothers in reducing infant caries experience is highlighted in a systematic review by Leong *et al*.^24^ Indeed current health promotion strategies enabling people to increase control over their own health, through social and environmental interventions holds 3 key elements: good governance for health, health literacy and healthy cities.^25^

Our study shows that one in three expectant mothers had never had oral health advice. Most of our participants did not know that milk, dried fruits and fruit juices can be cariogenic. Despite knowing that toothbrushing, dental floss and sugar-free chewing gum prevents dental caries, they did not know about fluoride varnish or other fluoride supplements. Those women who had children already knew more about the timing of starting toothbrushing and they had already decided which toothpaste to buy for their child. Although there was consensus that sugary foods cause dental caries (88%), very few knew that breast milk can cause caries. This is similar to another study where only a third of the mothers knew that prolonged breastfeeding can lead to tooth decay.^26^ Given the benefits of breastfeeding, the practice should be strongly encouraged. Dental professionals should promote adequate oral hygiene as soon as the first tooth erupts and inform that night-time prolonged feeds may cause ECC.^15,16^

This sample of pregnant women correlated well with the national average in age and ethnicity diversity.^27^ Pregnant women’s perception of their oral health was similar to that of the general adult population and to other studies.^28–32^ There was a good knowledge of the role of fluoridated toothpastes in dental caries prevention, which correlated to other studies,^33^ Those women who already had children knew when to start toothbrushing and the correct toothpaste dose.^19,28^ Sugar-free chewing gums appear to have gained popularity, perhaps attributed to the recent increase in mass media coverage of its use and benefits.

In 2014, the Public Health Advisory Committee emphasised the importance of collaborating with families ‘to establish healthier dietary patterns (including sugar-free diet) for both oral and general health’.^34^ More than half of pregnant women in our sample reported that they welcomed the idea of receiving oral health information and advice. Similar findings were seen in a local sample of children with ECC.^19,35^ Upon interviewing a number of women regarding their pregnancies and oral knowledge, Buerlein *et al.*^36^ also found that the majority of participants were highly motivated to implement the advice received, but they also complained that they had not received the information early enough.^36^ This was also supported by Habashneh, where lack of timely information emerges as the culprit of limited knowledge.^37^ Many studies emphasize the gap in dental knowledge and practices related to oral care^38–40^ but equally lack of knowledge and oral health practices are often associated^41^ and poor oral knowledge is sought to influence self-care decisions.^42^

In the UK, since the introduction of the ‘5 a day’ advert for daily intake of fresh fruit and vegetables, the public have misunderstood that frequent consumption of dried fruit and fruit juices is healthy.^43^ The present study has shown that expectant mothers did not know that dried fruit and fruit juices are cariogenic. One can speculate that this might reflect the wider population and that the ambiguous message from the ‘NHS 5 a day—live well’ has contributed to this, since it depicts fresh, frozen, canned and dried fruit equally counting towards the ‘5 a day’.

A surprising finding in our study was that social media only formed a minute source of information for pregnant women, reinforcing the importance of one-to-one interactions with health care professionals. Indeed, a recent behavioural intervention in pregnant women with obesity efficiently improved the quality of their diet.^44^ A literature review conducted by the Saskatchewan Prevention Institute reports the ‘need for creative, consistent, and comprehensive communication strategies that promote oral health to women in accessible and timely manners.^45^

One of the limitations of our study was the sampling method; a convenience sample may have led to sampling bias. A future study should include interpreters. The use of a self-administered questionnaire relied on the direct responses of pregnant women. Neither their oral health nor their oral health behaviour was verified. Equally, a source of bias, since recall is not perfect, is intrinsic to studies based on participants’ self-reporting. Therefore, some women might have under reported some options or overestimated others (such as dental attendance and toothbrushing frequency). The variability in knowledge between the participants might have been a confounding influence due to socioeconomic status and education levels,^33^ employment status,^26^ self-efficacy and locus of control,^19^ none of which were measured in this study. Enquiring about the use of dental floss within the context of caries prevention is controversial, particularly in children. Cochrane Systematic Reviews on the topic in 2011 and 2013 report weak and unreliable evidence.^46,47^ A Cochrane Systematic Review in 2015 on the management of proximal caries lesions in primary and permanent teeth acknowledge it might avoid substance loss.^48^ Evaluating the benefit from flossing is thus complicated by it being a technique-sensitive intervention and the fact that people are not always truthful when reporting about their engagement in flossing behavior.^49^

Overall, this study has revealed a lack of oral health knowledge among pregnant women, even though they support receiving oral health information at this time. Although service provision, professional attitude and government policies promote the acquisition of behaviours, understanding the habits and acquired knowledge of a population constitute a key plank in delivering oral health. Additionally, involving participants and members of the public into commenting and developing research materials is essential to refine a project aims and outcomes and to offer the best health care (INVOLVE).^50^ The findings on this study can support the design of an integrated antenatal-oral care programme targeting the needs of expectant mothers and their offspring to address the benefit of fluoride varnish, correct fluoride dose and timing of toothbrushing, cariogenicity of dried fruit, fruit juices, as well as avoiding prolonged milk feeding during the night after weaning.

## Figures and Tables

**Table 1:** Demographics of the participant (*participant declined to answer)

	n *(%)*
*Age (years)*	
20–24	9 (7.8)
25–29	33(28.7)
30–34	38 (33)
35–39	16 (13.9)
40–44	2 (1.7)
Missing	17 (14.8)*
Mean (s.d.), median, range	30.4 (4.67), 30.5, 20–43
	
*Number of children*	
0	58 (50.5)
1	34 (29.5)
2	17 (14.8)
3	3 (2.6)
4	2 (1.7)
Missing	1*
	
*Ages of children (years)*	
1–3	35 (41.2)
4–6	19 (22.4)
7–10	15 (17.6)
11–15	16 (18.8)
Mean (s.d.), median, range	6.2 (4.8), 5.25, 1–15

**Table 2:** Weekly frequency of cariogenic food and drinks

	n *(%)*
*Number of times ate biscuits, sweets or chocolates*	
Frequent	63 (54.8)
Occasional	50 (43.5)
Missing data	2 (1.7)
	
*Number of times drank fizzy drinks, fruit juices or soft drinks*	
Frequent	50 (43.5)
Occasional	62 (53.9)
Missing data	3 (2.6)

**Table 3:** Oral health practices (daily) and dental attendance (per year)

	n *(%)*
*Toothbrushing frequency*	
Minimum twice a day	88 (76.5)
Once daily to none	26 (22.6)
Missing data	1 (0.9)
	
*Oral hygiene practices*	
Toothbrushing	113 (98.3)
Fluoride supplements	1 (0.9)
Mouthwash	52 (45.2)
Regular dental visits	21 (18.3)
Sugar-free chewing gum	20 (17.4)
Dental floss	44 (38.3)
Missing data	1 (0.9)
	
*Dental attendance*	
Regular	70 (60.8)
Irregular	44 (38.3)
Missing data	1 (0.9)
